# Supplementary figures and images for: Appropriateness of indirect markers of muscle damage following lower limbs eccentric-biased exercises: A systematic review with meta-analysis
Source: PLoS One. 2022 Jul 14;17(7):e0271233. doi: 10.1371/journal.pone.0271233 (PMC9282447; doi:10.1371/journal.pone.0271233)

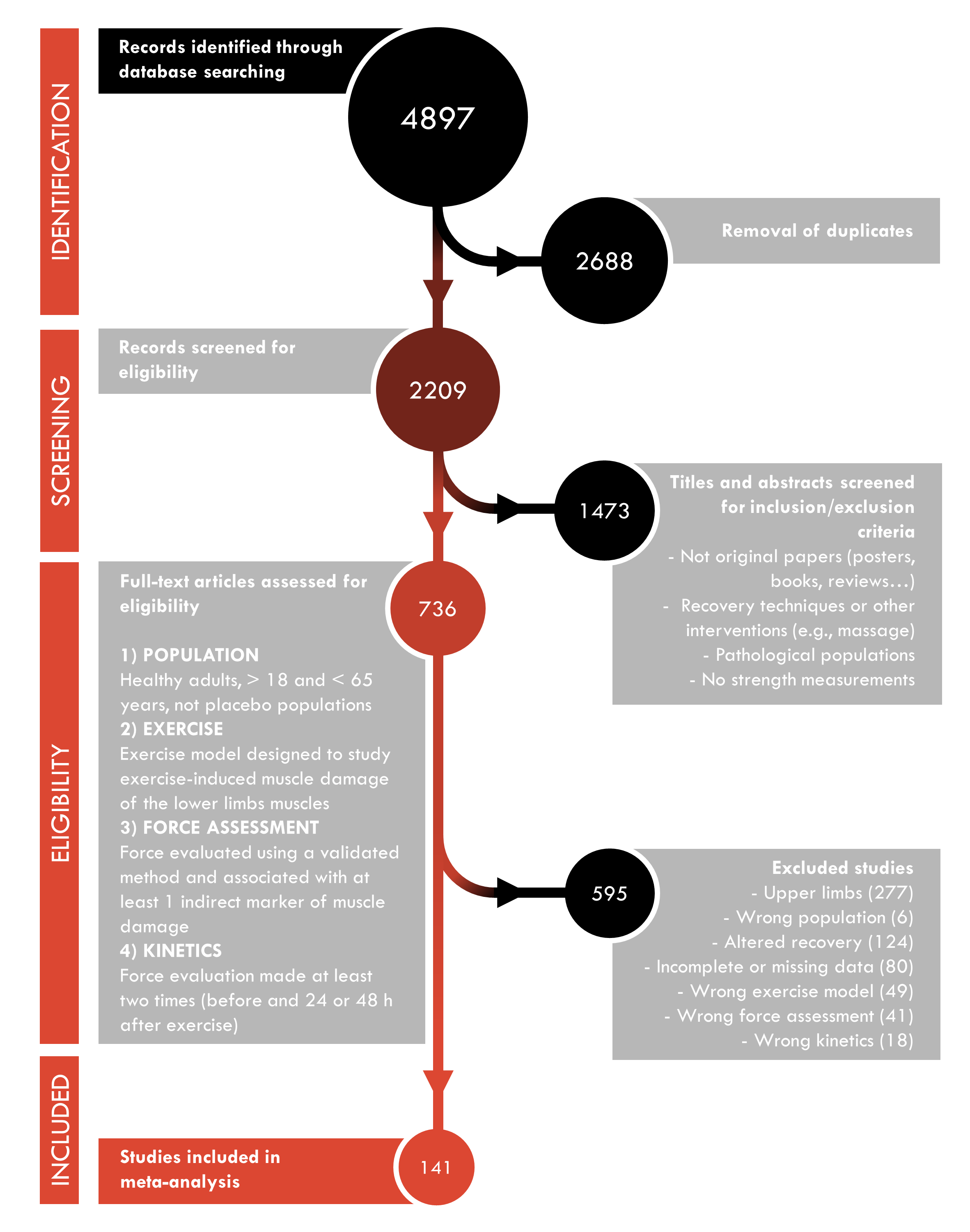

Supplement: S1 Fig — (TIF) [file pone.0271233.s002.tif]
